# Loose Coupling of Wearable-Based INSs with Automatic Heading Evaluation

**DOI:** 10.3390/s17112534

**Published:** 2017-11-03

**Authors:** Dina Bousdar Ahmed, Estefania Munoz Diaz

**Affiliations:** Institute of Communications and Navigation, German Aerospace Centre (DLR), Oberpfaffenhofen, 82234 Wessling, Germany; estefania.munoz@dlr.de

**Keywords:** wearable devices, position tracking, INS, IMU, pedestrian navigation, fusion, drift, quality factor, heading quality, inertial navigation

## Abstract

Position tracking of pedestrians by means of inertial sensors is a highly explored field of research. In fact, there are already many approaches to implement inertial navigation systems (INSs). However, most of them use a single inertial measurement unit (IMU) attached to the pedestrian’s body. Since wearable-devices will be given items in the future, this work explores the implementation of an INS using two wearable-based IMUs. A loosely coupled approach is proposed to combine the outputs of wearable-based INSs. The latter are based on a pocket-mounted IMU and a foot-mounted IMU. The loosely coupled fusion combines the output of the two INSs not only when these outputs are least erroneous, but also automatically favoring the best output. This approach is named smart update. The main challenge is determining the quality of the heading estimation of each INS, which changes every time. In order to address this, a novel concept to determine the quality of the heading estimation is presented. This concept is subject to a patent application. The results show that the position error rate of the loosely coupled fusion is 10 cm/s better than either the foot INS’s or pocket INS’s error rate in 95% of the cases.

## 1. Introduction

A wearable device, or wearable, is a device carried on the human body either by integrating it in the clothes, e.g., in the socks [1], or by attaching it to the body, e.g., a smart watch. The purposes of these devices are varied. In fact, the applications range from fitness trackers and health monitoring to context awareness, time management, communication, etc. According to Forbes [2], the market of wearables is exploiting. CCS Insight [3], a data analytic provider, predicts that around 411 million wearables will be used by 2020, and they will be worth a value of $34.2 billion. The economical impact of wearables is not the only factor worth considering. Wearables are being used increasingly outside the personal time. For instance, companies are testing the use of wearables for real-time employee communication, security access or employee time management [4].

The variety of wearables [5] makes available to researchers and developers multiple types of information. The latter can be classified in two main types: contextual information and user information. On the one hand, contextual information is collected with technology such as radio-frequency identification (RFID), Bluetooth, WiFi, etc. Such technologies provide information about the context surrounding the user, e.g., RFID tags or Bluetooth beacons placed on strategic known points. On the other hand, user information is collected through sensors such as IMUs, barometers, pressure sensors, etc. These sensors provide information about the user’s biological activity. For example, the shoe insert developed by 3Lab Footlogger [6] uses pressure sensors to implement activity tracking, fall detection, early detection of diseases like dementia or spinal disease, monitor safety of senior citizens, coaching of athletes or kids, etc.

All in all, the sensors embedded in wearable devices allow for the implementation of multiple applications, being localization one of them. There are multiple types of navigation systems given the technology available in wearable devices. GPS-based navigation is an option if the wearable can afford such a technology [5]. Nevertheless, low satellite-signal reception in urban canyons hinder the use of GPS-based solutions in such scenarios. Needless to say that the use of GPS-based systems in indoor environments is unfeasible. Alternative technologies and approaches have arisen for scenarios like urban canyons and indoor environments. Frequently, two or more technologies are combined with the goal of profiting from the advantages of each one. For instance, the smartphone-based navigation system in [7] combines WiFi, inertial measurements, i.e., acceleration and turn rate, and landmark detection. The system in [8] combines the inertial and the magnetic field measurements collected from a foot-mounted device. An alternative approach is presented in [9], where the inertial and Earth magnetic field measurements of a foot-mounted device are combined. Further approaches with inertial technology are possible. In [10], the position estimates of a pocket-mounted INS are combined with an RSSI-based navigation solution.

The focus of this work is pedestrian localization with inertial technology. IMUs are widely integrated within wearable devices, and therefore they are available for the implementation of INSs. Common approaches of INSs use one IMU mounted somewhere on the body. For instance, the foot [8,9]. However, in mass-market applications, users might be reluctant to use a foot-mounted device for diverse reasons. Therefore, other approaches have been explored. These approaches range from smartphone-based INS [11], to navigation with a wrist-mounted IMU [12], chest-mounted [13] or a pocket-mounted IMU [14] among others.

Whereas approaches for navigation with one IMU are varied, approaches using multiple IMUs are less numerous in comparison. The reason is that their main disadvantage, i.e., the drift of the heading over time, is addressed by combining the INS with other technologies. For instance, inertial technology and WiFi [7], inertial technology with RFID [15], etc. Nevertheless, the use of multiple inertial measurement unit (multi-IMU) has proven to be successful for either calibrating INSs or improving the heading drift.

Multi-IMU systems for INSs calibration develop methods to estimate the parameters of step length models that are specific to each pedestrian. For that purpose, a foot-mounted IMU [16] or multiple foot-mounted IMUs [17] can be used to estimate the step length of a user. The estimated step length can be used to compute the parameters of the step length model specific to a pedestrian [16,17].

Regarding multi-IMU systems for heading drift reduction, Bancroft et al. [18] explore different algorithms to combine up to five IMUs mounted on a single foot. Another option is to create an IMU array to obtain a better performance than a single IMU [19]. Skog et al. [20] use two foot-mounted IMUs, one on each foot, to implement two independent INSs. Then, a constraint of maximum separation between the two position estimates is applied to limit the growing drift in the heading. In [21], four IMUs are used to implement a navigation system for a virtual reality application. The orientation estimation of the head-mounted IMU is aided by the chest-mounted and the feet-mounted IMUs.

Multi-IMU navigation faces different challenges, being the heading drift the most important. The heading drift is caused mainly due to the bias in the turn rate measurement of the IMU. We identify another challenge in the heading estimation: the inability to know how much drift a system presents at a specific time. That is, it is a fact that the heading estimated by an INS drifts over time [22]. However, it is also a fact that the drift is random. For instance, let us consider one INS operating at different days but under similar conditions and with the same IMU. It would be expected that the heading drifts approximately the same quantity in either day. However, the randomness in the drift does not allow to make such statement, which is supported by experimental tests [23].

### 1.1. Challenges of Multi-IMU Navigation for Pedestrians

The challenges in the development of multi-IMU navigation systems arise due to the possible approaches to combine the sources of information. We envision two main approaches to combine the information: loose coupling and tight coupling. Table 1 summarizes the generic aspects of the identified multi-IMU navigation approaches.

**Figure 1:**
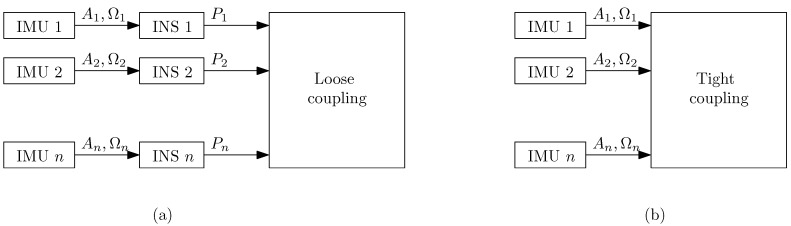
Block diagram of the (**a**) loose coupling approach and (**b**) tight coupling approach. $A_{i}$, $\Omega_{i}$ and $P_{i}$ represent the acceleration vector, turn rate vector and position vector respectively.

*Loose coupling* refers to having independent INSs and combining their outputs to estimate a single position of the pedestrian, see Figure 1a. Two generic challenges are identified, Table 1: *how* and *when*. That is, how to do the combination of the output of the INSs and when to do it. The *how* can be a deterministic approach or a probabilistic approach. The *when* needs to consider the characteristics of each INSs. For example, let us consider an INS based on a strapdown approach and an INS based on the step-length-and-heading estimation approach. The strapdown approach provides a new position estimate with each new inertial measurement. In contrast, the step-length-and-heading estimation approach provides a new position estimate only after a step is detected. The frequency of inertial measurements is higher than the pedestrian’s steps. Thus, the output frequency of the INS based on strapdown is higher than the output frequency of the INS based on step-length-and-heading estimation. As a result, the frequencies of the INSs’ output need to be considered in the loose coupling approach. Another factor to consider is whether the INS incorporates special events of the human gait cycle in the position estimation. For example, the stance phase detection in the strapdown approach [24]. The reason is that, such events are used to improve the position estimation. Therefore, a smart fusion might consider the position estimates only when they are least erroneous. A loose coupling of INSs has the advantage of modularity because it uses the output of independent INSs. The redundancy of independent INSs helps to the robustness of the overall INS, which is an approach frequently followed in other areas, e.g., in communications or programming [25]. Nevertheless, redundancy implies having duplicated functionality, which could be a restrictive factor for certain applications.

*Tight coupling* refers to using the inertial measurements of each IMU in a single algorithm without being previously processed by an INS, see Figure 1b. The main challenge identified is *how*, see Table 1. That is, how to do the fusion of the raw inertial measurements. Multiple approaches are possible, yet all of them should get advantage of the fact that the inertial measurements are from the same person. That is, constraints can be defined and incorporated in a single probabilistic filter, similar to [26]. A tight coupling approach should be efficient in time and computational resources. The reason is that the raw measurements are combined from the beginning, without additional processing as opposed to the loose coupling approach.

### 1.2. Objective of This Work

The objective of this work is to combine the information from two body-mounted IMUs following a loose coupling approach, see Figure 1a. We set three boundary conditions to the challenge that this work addresses.

**Figure 2:**
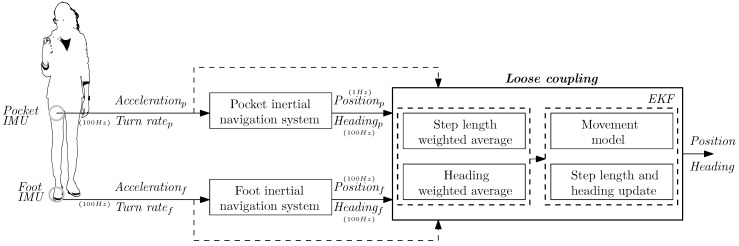
Loose coupling approach based on a pocket-mounted IMU and a foot-mounted IMU.

The first boundary condition is the body location of the IMUs. The IMUs used in this work are located on the upper-front part of the foot and on the upper thigh of the same leg, see Figure 2. Each of these body locations has advantages that are exploited in their respective INSs.

The second boundary condition is the INSs, which are specific to the body location of the IMU. The foot-mounted INS used in this work is described in [27] and references therein. This INS follows the strapdown approach to estimate the user’s position. The stance phase of the foot is detected to apply zero-velocity updates (ZUPTs), which correct the position estimates. The thigh-mounted INS used in this work is described in [14]. This INS follows the step-length-and-heading estimation approach. The opening angle, or pitch, of the leg is used to estimate the user’s step length. In the reminder of this paper, the thigh-mounted INS will be referred to as pocket-mounted INS. The detailed description of the foot-mounted INS and pocket-mounted INS is out of the scope of this work. For further details, the reader is referred to the aforementioned papers and references therein.

The last boundary condition to define is the approach of the loosely coupled fusion. The approach to the loose coupling refers to the general guideline on how to perform the fusion. Table 1 identifies, under the *how* branch, two main approaches: deterministic and probabilistic. A simple deterministic approach is to average the position estimate generated by each INS. In this work, a probabilistic approach based on an exteded Kalman filter (EKF) is followed. The reason is that these filters are suited for the fusion of multiple sources of information to estimate a set of parameters or states. Furthermore, this work attempts to combine the systems by exploiting their best qualities. The definition of the approach requires determining the parameters, or states, to be estimated by the loose coupling fusion. In this work, the states are defined to be the 3D position and the heading of the user.

All in all, the goal of this work can be described as follows: the proposal of a loosely coupled fusion that combines the information from a foot-mounted INS and a pocket-mounted INS in a probabilistic fashion.

## 2. Proposed Methods

The goal of this work is to propose a loose coupling system to estimate the position of a pedestrian. This system and its subsystems are described in the following sections.

### 2.1. Proposed Loose Coupling Approach

The loose coupling system proposed in this work is depicted in Figure 2. According to the loose coupling in Figure 1, the inertial measurements from the pocket-mounted and foot-mounted IMUs are processed independently by each INS. The outputs of the latter, i.e., position and heading, are fed to the loose coupling system. The loose coupling system combines the information in two steps. The first one estimates a weighted average of the step length and heading of each input INS, which is explained in Section 2.2 and Section 2.3. The second step is an EKF that implements a movement model in the prediction step and updates the states with the step length and heading estimated in the first step of the loose coupling. The filter is explained in Section 2.4. The EKF produces a single position and heading estimation. The goal is that the final estimated position and heading are better than the position and heading estimations of the foot INS and pocket INS.

The loose coupling system must consider two aspects, which are already outlined in Table 1. These items are: the output frequency of each INS and the instant when the outputs of the INSs are least erroneous.

Firstly, the output frequency of the INSs depends on the pedestrian dead-reckoning algorithm that each INS implements. In the case of the foot INS, the strapdown approach is followed [27]. Every new turn rate measurement is used to obtain a new orientation estimation, which includes the heading estimation. Likewise, every new acceleration measurement is double-integrated to obtain a new position sample. Therefore, the frequency of both the position and heading estimation of the foot INS is the same as the frequency of the input. In this case, the input frequency to the foot INS is 100 Hz. In the case of the pocket INS, the step-length-and-heading estimation approach is followed [14]. Like with the foot INS, each turn rate measurement at the input of the pocket INS is used to produce a new orientation estimation. Therefore, the frequency of the pocket-INS’s heading is the same as the frequency at the input of the pocket INS, which is 100 Hz. In contrast, the position of the pocket INS can be estimated only when a stride event is detected. This event occurs approximately once very second, i.e., with 1 Hz frequency. The knowledge of the frequencies is necessary because multiple fusion approaches can be defined depending on them.

Secondly, the instant when the output of the INSs are least erroneous, i.e., they are most accurate, depends on which phase of the gait cycle are included in the INS. Both the foot INS and the pocket INS incorporate phases of the gait cycle in the estimation of the position. Upon occurrence of these events, the estimations of each INS are most accurate. Therefore, we decided to combine the outputs of the INSs upon detection of these events. The combination proposed in this work is named smart update, and it will be explained in detail in the next section.

### 2.2. Smart Update

Smart update refers to the fact of combining the output of two or more INSs when these outputs are most accurate and favouring the least erroneous one. The output of an INS is most accurate during a certain phase of the gait cycle. This phase might coincide or not with that of another INS. In the following, we explain the events of the gait cycle that the foot INS and the pocket INS use, as well as which role these phases play in the loose coupling.

**Figure 3:**
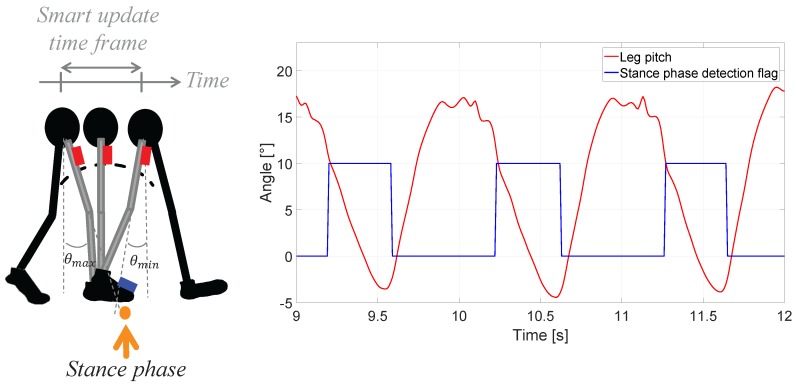
(**Left**) Gait event: stance phase. While the foot is in stance phase, the thigh’s pitch changes from its maximum to it minimum angle while walking; (**Right**) Leg’s pitch estimated and flag for the detection of the foot’s stance phase during three stance phases. The leg’s pitch is estimated by the pocket Inertial Navigation System (INS) and the stance phase flag is estimated by the foot INS.

The foot INS detects the stance phases of the foot, i.e., when the foot is in contact with the ground, see Figure 3. During the stance phase, the foot is static and therefore its velocity is zero. This information can be incorporated in the foot INS to improve the position estimation [27].

The pocket INS detects strides in order to estimate the step length. A stride is detected when the pitch of the leg where the IMU is mounted is maximum, see Figure 3. Upon step length estimation, the pocket INS can produce a new position estimate.

As it can be seen in Figure 3, the maximum pitch of the thigh occurs at the same time as the stance phase of the foot. That is, the gait events at which the pocket INS and the foot INS produce the most accurate position estimate occur simultaneously. Therefore, the output of both INSs can be combined when both events are detected. Furthermore, both events happen once in every gait cycle, i.e., approximately with 1 Hz frequency. Therefore, the combination of both the step length and heading is done at 1 Hz. The smart update is implemented in the update stage of the EKF of the loose coupling.

So far, it has been determined when to combine the information of both INSs. The next step is to determine how to combine the information. A possible approach is to compute a weighted average of the information, i.e., step length and heading, see Figure 2. Although a weighted average might seem like a simple approach, it requires the estimation of the weights for the foot INS’s and pocket INS’s step length and heading. Following the smart update principle, the estimation of these weights must be such that the most accurate information at the input of the loose coupling is favoured. The estimation of these weights is presented in the next sections.

#### 2.2.1. Smart Update of the Step Length

The smart update of the step length takes place in the update stage of the EKF in Figure 2. This update requires a single step length, which must be computed out of the two available information at the input of the loose coupling: the position estimation of the pocket INS and the foot INS.

Figure 2 indicates that the step length (*s*) is computed as a weighted average, which can be expressed as:

(1) $$s=w_{s,f}\;\cdot \;s_{f}+w_{s,p}\;\cdot \;s_{p},$$

where $s_{f}$ and $s_{p}$ are the step lengths estimated by the foot INS and the pocket INS, respectively. These step lengths are computed from the position estimation of each INS. $w_{s,f}$ and $w_{s,p}$ are the weights of the foot INS’s step length and the pocket INS’s step length respectively.

The smart update principle requires these weights to reflect how trustworthy the step length estimation of each INS is. Therefore, the estimation of these weights is done based on the result of a study conducted to characterize and compare the performance of the foot INS and the pocket INS here used [23].

The study showed that the foot INS has a better distance estimation than the pocket INS. This result is due to the fact that the pocket INS estimates the step length based on a model. The latter has to be adapted to each user for an optimal performance, whereas the foot does not use any models to estimate distances.

Based on this knowledge, the weights in (1) can be set to a fix value. This value has to be higher for the foot INS than for the pocket INS. For that purpose, the results of the study in [23] are considered. According to them, the foot INS has a root mean square error (RMSE) of 0.7 m, whereas the pocket INS’s RMSE is 1.2 m. Thus, the weight of the foot INS’s step length is computed as:

(2) $$w_{s,f}=\frac{\sigma_{f}^{-1}}{\sigma_{f}^{-1}+\sigma_{p}^{-1}},$$

where $\sigma_{f}^{-1}$ and $\sigma_{p}^{-1}$ are the RMSE of the foot INS and the pocket INS respectively. Similarly, the weight of the pocket INS’s step length is:

(3) $$w_{s,p}=\frac{\sigma_{p}^{-1}}{\sigma_{f}^{-1}+\sigma_{p}^{-1}}.$$

#### 2.2.2. Smart Update of the Heading

The smart update of the heading also takes place during the update of the EKF. Additionally, the heading (ψ) is also computed as the weighted average of the headings at the input of the loose coupling system, i.e.,

(4) $$\psi =w_{\psi ,f}\;\cdot \;\psi_{f}+w_{\psi ,p}\;\cdot \;\psi_{p},$$

where $\psi_{f}$ and $\psi_{p}$ are the heading estimations of the foot INS and the pocket INS respectively. $w_{\psi ,f}$ and $w_{\psi ,p}$ are the weights of the foot INS’s heading and the pocket INS’s heading respectively.

The difference of the smart update of the heading with respect to the smart update of the step length relies on how the weights $w_{\psi ,f}$ and $w_{\psi ,p}$ are computed. In this case, the study in [23] is also taken as a reference. The study states that no general statement about the heading estimation of the foot INS and the pocket INS can be made. That is, the quality of one INS’s heading varies with respect to the heading quality of the other INS. That is due to the random drift that affects the orientation estimation in inertial systems. The drift in the orientation estimation is caused due to the bias in the turn rate measurements. The bias depends on external conditions, such as temperature, and it might not be the same at different runs of the system. Furthermore, the drift is especially observable in the heading estimation of an INS. The reason is that the heading is not observable through the gravity, as opposed to the roll and pitch. The observability of the roll and pitch by measuring the gravity force allows for corrections of these two angles. However, the heading cannot profit from such a correction.

The consequence is that the heading estimation is disturbed by a drift whose effect cannot be determined a priori. Thus, the heading weights in (4) cannot be fixed, in contrast to the weights of the step length. In fact, the weights in (4) must be variable and computed online, i.e., the weights must be computed specifically for the heading estimations available at a certain time.

A novel approach, which determines the quality of the heading of an INS, is developed. This novel approach estimates, for the heading of each INS, a quality factor (*q*). The latter informs about the quality of the heading of an INS. The higher the quality factor, the less erroneous the heading, and thus, the higher the associated weight should be. Based on this quality factor, the heading weight of the foot INS can be computed as:

(5) $$w_{\psi ,f}=\frac{q_{f}}{q_{f}+q_{p}},$$

where $q_{f}$ and $q_{p}$ refer to the quality factor of the foot INS’s heading and the pocket INS’s heading respectively. Similarly, the weight of the pocket INS’s heading can be computed as:

(6) $$w_{\psi ,p}=\frac{q_{p}}{q_{f}+q_{p}},$$

The detailed explanation of the estimation of the quality factor is provided in the next section.

### 2.3. Quality Factor

The smart update approach attempts to combine the input data to a fusion system in the best way, i.e., favouring the least erroneous input at each time. Such an approach requires knowledge about the quality of the input data.

**Figure 4:**
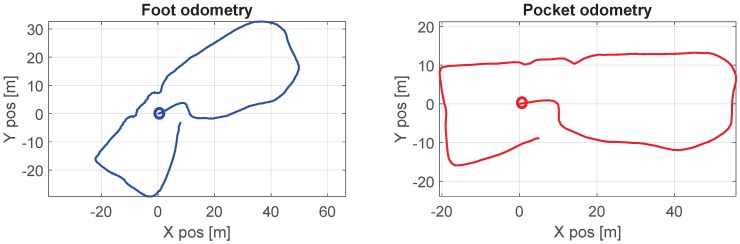
Odometry of a walk estimated by the foot INS (**left**) and the pocket INS (**right**) in Figure 2. The circles indicate the beginning of the walk. An observer cannot identify which INS has a better heading estimation without knowledge of the ground truth.

The challenge regarding the heading is to determine which INS is producing, at a certain time, the most accurate heading estimate. When prior information is available, determining which INS is estimating the heading better is immediate. On the contrary, i.e., if no information is available, making a statement on which heading is the least erroneous, out of two or more heading estimations, is not immediate. For example, let us consider the odometry estimations in Figure 4. Both odometries belong to the same walk but they were generated with different INSs, namely the two in Figure 2. It is clear that both estimations are different, and without any further information, it is not possible to state which odometry has the least erroneous heading estimation. The latter is the challenge that we attempt to approach.

#### 2.3.1. Method for the Estimation of the Quality Factor

In order to determine which INS produces a less erroneous heading estimation, the use of a quality factor is proposed. The quality factor is a value that indicates how accurate or erroneous the heading estimation of an INS is. The quality factor is not meant to be used standalone, but as a means of relative quality of different estimations of the same parameter.

**Figure 5:**
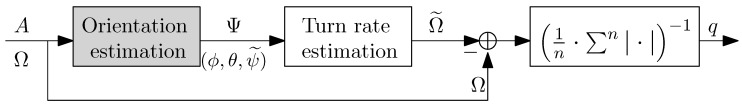
Estimation of the quality factor (*q*). The *Orientation estimation* block is part of any INS, and it implements a probabilistic filter. The heading estimated by this block is the one whose quality has to be assessed.

The estimation of the quality factor (*q*) is presented in Figure 5. In the latter, the shadowed *Orientation estimation* block represents the orientation estimation of an INS. The orientation estimation is assumed to be implemented by a probabilistic filter, e.g., a Kalman filter or some variation of it. The orientation estimation is represented by three angles, known as Euler angles: the roll (ϕ), the pitch (θ) and the yaw or heading (ψ).

The first step in the estimation of the quality factor (*q*) is to determine the turn rate vector ($\tilde{\Omega }$) that generates the roll (ϕ), pitch (θ) and drifting heading ($\tilde{\psi }$). For that purpose, the relationship between the turn rate vector and the Euler angles has to be determined. This relationship must consider the order in which the Euler angles were computed in the *Orientation estimation* block, see Figure 5. In this case, the Euler angles are estimated by applying three successive rotations around the *x*-axis, *y*-axis and *z*-axis. Each one by an amount of ϕ, θ and ψ degrees respectively. Under this consideration, the equations that relate the estimated turn rate vector in the sensor frame, $\tilde{\Omega }=\left({\tilde{\omega }}_{x},{\tilde{\omega }}_{y},{\tilde{\omega }}_{z}\right)$, with the estimated Euler angles, ($\phi ,\theta ,\tilde{\psi }$), are:

(7) $$\begin{array}{ccc} {\tilde{\omega }}_{x} & = & \dot{\phi }-\dot{\tilde{\psi }}\;\cdot \;\sin\left(\theta \right),\\ {\tilde{\omega }}_{y} & = & \dot{\theta }\;\cdot \;\cos\left(\phi \right)+\dot{\tilde{\psi }}\;\cdot \;\sin\left(\phi \right)\;\cdot \;\cos\left(\theta \right),\\ {\tilde{\omega }}_{z} & = & -\dot{\theta }\;\cdot \;\sin\left(\phi \right)+\dot{\tilde{\psi }}\;\cdot \;\cos\left(\phi \right)\;\cdot \;\cos\left(\theta \right), \end{array}$$

where $\dot{\phi }$, $\dot{\theta }$ and $\dot{\psi }$ are the roll-rate, pitch-rate and yaw-rate respectively. The equations in (7) are implemented in the *Turn rate estimation* block in Figure 5.

The next step is to compare the estimated turn rate vector ($\tilde{\Omega }$) and the true measured turn rate vector (Ω), see the subtraction operation in Figure 5. The assumption is that the roll (ϕ) and pitch (θ) estimations are error-free when compared to the estimated heading ($\tilde{\psi }$) [28]. The latter is mainly disturbed by the drift, which is caused by the bias of the gyroscopes. Therefore, when the estimated Euler angles are used to estimate the associated turn rate vector ($\tilde{\Omega }$), the drift that disturbs the heading estimate ($\tilde{\psi }$) also disturbs the estimated turn rate vector ($\tilde{\Omega }$). In fact, the larger the drift is the more different $\tilde{\Omega }$ and Ω will be and, thus, the lower the quality factor should be.

The average over *n* samples in Figure 5 is necessary because the drift is a low-frequency error. In this case, a window length of 10 s is used. Finally, the quality factor is estimated by inverting the averaged difference $|\Omega -\tilde{\Omega }|$.

The quality factor (*q*) is affected by two aspects. Firstly, the drift in the yaw which is represented by the noisy yaw-rate ($\dot{\tilde{\psi }}$) in (7). Secondly, the set of values of the roll (ϕ), roll-rate ($\dot{\phi }$), pitch (θ) and pitch-rate ($\dot{\theta }$). The latter set of values will determine how much the effect of the drift can be observed in each of the components of the difference vector $\left(\Omega -\tilde{\Omega }\right)$.

All in all, the quality factor allows to compare different heading estimations in order to know which one has least drift. The advantage of this method is that no ground truth information is required to estimate the quality factor. That is, the quality factor can be computed online and it requires only the estimated heading ($\tilde{\psi }$) and the measured turn rate (Ω) to determine which INS, out of two or more, has least drift.

**Figure 6:**
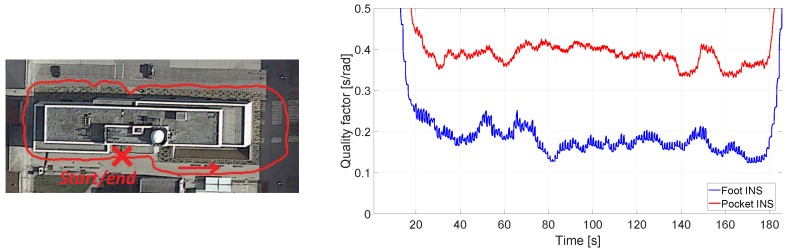
(**Left**) Ground truth of the walk of Figure 4; (**Right**) Quality factor estimated for each of the odometries of Figure 4.

Let us consider again the case depicted in Figure 4. It was previously indicated that, without prior knowledge, it is not possible to state which INS has a better heading estimation. In using the method proposed in Figure 5, the quality factor estimated should indicate which INS has a better heading estimation. Figure 6 presents the ground truth of the walk in Figure 4. The pocket INS in Figure 4 is generating, qualitatively according to the ground truth, a better odometry than the foot INS. This is supported by the quality factors plotted in Figure 6. The quality factor of the pocket INS is higher than the foot INS’s heading estimation. That is, the pocket INS is less erroneous than the foot INS regarding the heading estimation of this walk. The quality factor can then be used to weight the heading estimation of each INS through Equations (5) and (6).

#### 2.3.2. Validation of the Method to Estimate the Quality Factor

**Figure 7:**
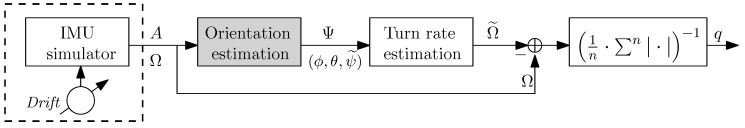
Simulation set up to verify the methodology to compute the quality factor. The drift generator can be configured as desired.

The methodology proposed in Figure 5 is validated through simulations. The validation has two main objectives. The first one is to show that the quality factor is the same when the heading estimations of different INSs have the same quality. The second one is to show that, if a heading estimation is more correct than another one, the quality factor is higher for the former than the latter. For that purpose, the configuration in Figure 7 has been used.

The IMU simulator generates acceleration and turn rate signals disturbed by random walk and by constant bias. As previously mentioned, the bias is the major contributor to the drift of the heading. The values for the random walk are chosen according to those of the Xsens wireless IMU, the MTw. The latter is the IMU used in the experiments presented in Section 3. The bias can be set to a constant value or a variable value.

A set of inertial measurements from a still IMU are generated with a constant bias on the *z*-axis. The constant bias takes values from 0${}^{\circ }$/s to 1${}^{\circ }$/s. These inertial measurements were processed with two different *Orientation estimation* blocks, those of the pocket INS and the foot INS respectively, see Figure 2. The heading estimation ($\tilde{\psi }$) of each block is used to estimate the associated quality factor.

**Figure 8:**
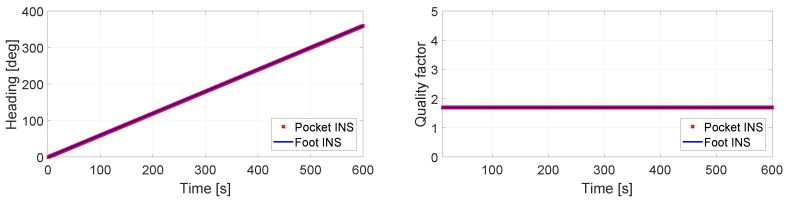
(**Left**) Heading estimation of the pocket INS and the foot INS of a still IMU with 0.6${}^{\circ }$/s constant bias in the *z*-axis of the turn rate measurements; (**Right**) Quality factor estimation of the heading estimations.

The results for a constant drift of 0.6${}^{\circ }$/s are presented in Figure 8. The latter shows that the heading estimation of both INS is the same. Moreover, in a time frame of 10 min and with a still IMU, the total drift is 360${}^{\circ }$. For these heading estimations, the associated quality factor is the same, as shown in the right plot of Figure 8. As previously mentioned, the quality factor is meant to be used as a measurement of relative quality. In the case of Figure 8, the interpretation is that both heading estimations have the same quality.

**Figure 9:**
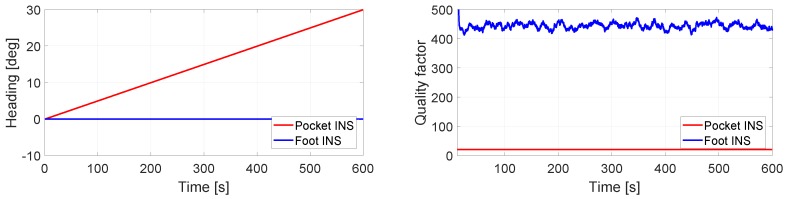
(**Left**) Heading estimation of the pocket INS and the foot INS of a still IMU. The turn rate measurements to the pocket INS have a 0.05${}^{\circ }$/s constant bias in the *z*-axis. The turn rate measurements to the foot INS have no bias; (**Right**) Quality factor estimation of the heading estimations.

Figure 9 presents the case where the turn rate measurements to the foot INS have no bias, whereas the turn rate measurements to the pocket INS are disturbed by a constant bias of 0.05${}^{\circ }$/s in the *z*-axis. Since the IMU is still during the 10 min of simulated data, the heading estimation should be 0${}^{\circ }$ at all times. The left plot in Figure 9 shows that the foot INS estimates correctly the heading, whereas the pocket INS’s heading estimation drifts. This behavior can be seen in the quality factor. The latter is significantly higher for the foot INS than the pocket INS, see right plot of Figure 9. Thus, the quality factor indicates that the foot INS’s heading estimation is better than the pocket INS’s heading estimation.

**Figure 10:**
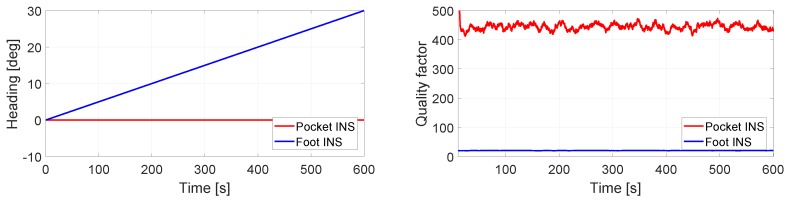
(**Left**) Heading estimation of the pocket INS and the foot INS of a still IMU. The turn rate measurements to the pocket INS have no bias. The turn rate measurements to the foot INS have a 0.05${}^{\circ }$/s constant bias in the *z*-axis; (**Right**) Quality factor estimation of the heading estimations.

Figure 10 presents the opposite case to Figure 9. The turn rate measurements to the foot INS are disturbed by a 0.05${}^{\circ }$/s constant bias on the *z*-axis. The turn rate measurements to the pocket are not disturbed by bias. Figure 9 and Figure 10 show that the behavior of the quality factor is equivalent, even if the heading under evaluation is estimated by different INSs.

**Figure 11:**
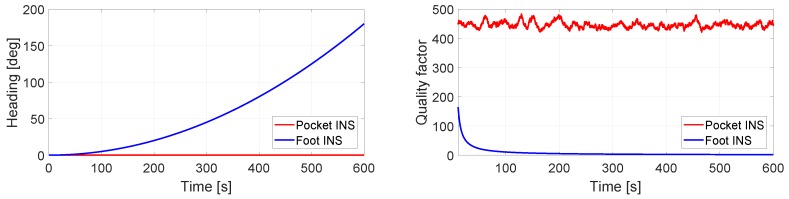
(**Left**) Heading estimation of the pocket INS and the foot INS of a still IMU. The turn rate measurements to the pocket INS have no bias. The turn rate measurements to the foot INS have a changing bias in the *z*-axis; (**Right**) Quality factor estimation of the heading estimations.

Figure 11 presents the case where the turn rate measurements to the pocket INS are not disturbed by bias. The turn rate measurements to the foot INS are disturbed by bias in the *z*-axis. In this case, the bias in the *z*-axis is not constant but variable. In fact, it increases at a rate of 0.05 ${}^{\circ }/s$/*s*. The changing bias leads to a heading estimation that does not drift linearly, but quadratically over time. Despite the behavior of the bias, the quality factor behaves as expected. That is, the quality factor indicates that the pocket INS’s heading estimation is better than the foot INS’s, see the right plot of Figure 11.

### 2.4. Extended Kalman Filter

This section describes the EKF in Figure 2. The EKF is a modification of the well-known Kalman filter for non-linear models [29]. The states of the EKF (*X*) are chosen to be the 3D-position of the user and the user’s heading:

(8) $$X_{k}={\left(x_{k},y_{k},z_{k},\psi_{k}\right)}^{T},$$

where $\left(x_{k},y_{k},z_{k}\right)$ is the user’s 3D-position and $\psi_{k}$ is the user’s heading at the *k*-th time. The states are propagated according to ${\hat{X}}_{k}=g\left(X_{k-1},u_{k}\right)$ which follows the equation:

(9) $${\hat{X}}_{k}=X_{k-1}+{\left(s_{k}\;\cdot \;\cos\left(\psi_{k-1}\right),s_{k}\;\cdot \;\sin\left(\psi_{k-1}\right),0,0\right)}^{T},$$

where $s_{k}$ is the step length at the *k*-th time. The step length $s_{k}$ is a fixed value which is estimated assuming that the user moves at 1 m/s and that the EKF predicts the states at 100 Hz. Therefore, the resulting step length ($s_{k}$) used in (9) is 1 cm. The Jacobian matrix $G_{k}$ of the process model is given by:

(10) $$G_{k}=\left(\begin{array}{cccc} 1 & 0 & 0 & -s_{k}\;\cdot \;\sin\left(\psi_{k-1}\right)\\ 0 & 1 & 0 & s_{k}\;\cdot \;\cos\left(\psi_{k-1}\right)\\ 0 & 0 & 1 & 0\\ 0 & 0 & 0 & 1 \end{array}\right).$$

The propagated states ${\hat{X}}_{k}$ have an associated covariance matrix ${\hat{P}}_{k}$ which is computed according to:

(11) $${\hat{P}}_{k}=G_{k}\;\cdot \;P_{k-1}\;\cdot \;G_{k}^{T}+Q,$$

where $P_{k-1}$ is the covariance matrix of the state estimation $X_{k-1}$ and *Q* is the covariance matrix of the process model noise.

The measurements used in the update stage of the filter are the user’s step length ($s_{k}^{z}$), the vertical displacement ($v_{k}^{z}$) and the user’s heading ($\psi_{k}^{z}$), i.e., the measurement vector ($Z_{k}$) can be composed as:

(12) $$Z_{k}={\left(s_{k}^{z},v_{k}^{z},\psi_{k}^{z}\right)}^{T}.$$

The step length and heading measurements are computed according to (1) and (4) respectively. In this work, only 2D-walks are considered so the vertical displacement $v_{k}^{z}$ is set to 0.

The measurement update is performed, as explained in Section 2.2, under the smart update principle. For the INSs used in this work, the outputs are least erroneous when two events occur: the stance phase of the foot and the maximum aperture of the leg. These two events overlap in time, as shown in Figure 3. Upon detection of these two events, which occur approximately with 1 Hz frequency, the states of the EKF are updated. The measurement model ${\hat{Z}}_{k}=h\left(X_{k}\right)$ for the update stage is as follows:

(13) $${\hat{Z}}_{k}={\left(\sqrt{{\left({\hat{x}}_{k}-x_{k-j}\right)}^{2}+{\left({\hat{y}}_{k}-y_{k-j}\right)}^{2}},{\hat{z}}_{k}-z_{k-j},{\hat{\psi }}_{k}\right)}^{T},$$

where ${\hat{Z}}_{k}$ is the estimated measurement vector. The subindex k−j refers to the time of the last measurement update. The measurement model has the following Jacobian matrix:

(14) $$H_{k}=\left(\begin{array}{cccc} H_{11,k} & H_{12,k} & 0 & 0\\ 0 & 0 & 1 & 0\\ 0 & 0 & 0 & 1 \end{array}\right),$$

where $H_{11,k}$ and $H_{12,k}$ follow the next equations:

(15) $$\begin{array}{ccc} H_{11,k} & = & \left({\hat{x}}_{k}-x_{k-j}\right)\;\cdot \;{\left({\left({\hat{x}}_{k}-x_{k-j}\right)}^{2}+{\left({\hat{y}}_{k}-y_{k-j}\right)}^{2}\right)}^{-1/2}, \end{array}$$

(16) $$\begin{array}{ccc} H_{12,k} & = & \left({\hat{y}}_{k}-y_{k-j}\right)\;\cdot \;{\left({\left({\hat{x}}_{k}-x_{k-j}\right)}^{2}+{\left({\hat{y}}_{k}-y_{k-j}\right)}^{2}\right)}^{-1/2}. \end{array}$$

Finally, the Kalman gain and update of the states follow the standard form of an EKF:

(17) $$\begin{array}{ccc} K_{k} & = & {\hat{P}}_{k}\;\cdot \;H_{k}^{T}\;\cdot \;{\left(H_{k}\;\cdot \;{\hat{P}}_{k}\;\cdot \;H_{k}^{T}+R\right)}^{-1}, \end{array}$$

(18) $$\begin{array}{ccc} X_{k} & = & {\hat{X}}_{k}+K_{k}\;\cdot \;\left(Z_{k}-{\hat{Z}}_{k}\right), \end{array}$$

(19) $$\begin{array}{ccc} P_{k} & = & \left(I-K_{k}\;\cdot \;H_{k}\right)\;\cdot \;{\hat{P}}_{k}, \end{array}$$

where *R* is the covariance matrix of the measurement model noise, $X_{k}$ is the updated states vector and $P_{k}$ is the updated covariance matrix of the states vector.

## 3. Evaluation

This section presents the results of evaluating the fusion method in Figure 2. These results are compared to those of the foot INS and the pocket INS. For that purpose, the evaluation methodology is described in the first place. Secondly, the results of the evaluation are presented and discussed.

### 3.1. Evaluation Methodology

The evaluation methodology was already presented in [30]. The evaluation methodology is based on ground truth points (GTPs), i.e., points whose location is known accurately. These GTPs are visited during the walks, and the inertial measurements from the latter are processed with the INSs. The INSs are then evaluated by comparing the true position and the estimated position of the GTPs.

The GTPs in [30] are distributed in an area of 14,380 $m^{2}$ approximately, and they account to a total of 69. The accurate location of the GTPs was measured with a tachymeter. The data set recorded in [30], which is made public by the authors, is summarized in Table 2.

**Figure 12:**
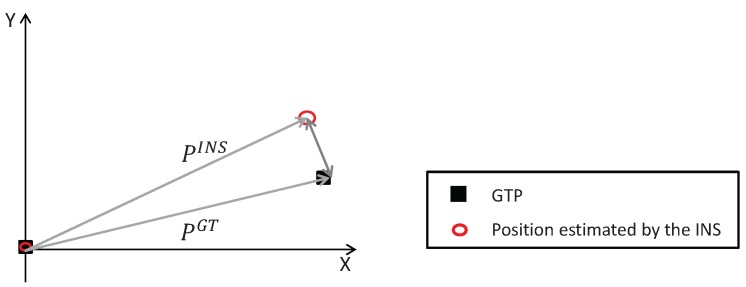
Representation of the position error between the true position of a GTP ($P^{GT}$) and its estimated position ($P^{INS}$).

The metric used to evaluate the INSs is the position error rate ($e_{P}$). The position error between the true position of a GTP and its estimated position is represented in Figure 12. The position error rate ($e_{P}$) is then computed by normalizing the position error by the elapsed time ($t_{k}$):

(20) $$e_{P}\left(k\right)=\frac{|P^{\mathrm{GT}}\left(k\right)-P^{\mathrm{INS}}\left(k\right)|}{t_{k}}.$$

Given that the average user makes one stride every second, the position error rate ($e_{P}$) can be seen as the error that the user makes in each stride.

### 3.2. Results

Table 3 presents the results of evaluating the foot INS, the pocket INS and the loosely coupled fusion with the experiments summarized in Table 2.

### 3.3. Discussion

The results in Table 3 show that, in average, the foot INS has a slightly smaller error per stride than the pocket INS. In turn, the pocket INS has a more uniform behavior than the foot INS, which can be appreciated by the smaller σ of the pocket INS than the foot INS’s σ.

The statistics of the fusion presented in Table 3 indicate that the method proposed in this work is, in average, as good as the foot INS. However, the fusion improves the standard deviation of the foot INS. That is, the fusion has a behaviour as uniform as the pocket INS’s. This result is encouraging since the fusion appears to profit from the advantages of each INS, as it is attempted with the system proposed in Figure 2.

**Figure 13:**
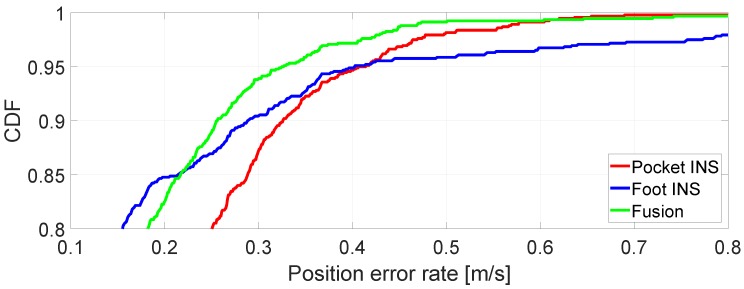
Cumulative distribution function (CDF) of the position error rate.

Figure 13 presents the cumulative distribution function (CDF) of the position error rate of each INS under evaluation. The CDF indicates the probability that the position error rate is equal to or smaller than a given value. Figure 13 shows that in approximately 85% of the cases, the position error rate of both the foot INS and the fusion method is equal to or below 22 cm/s, i.e., 22 cm per stride. If a higher probability is considered, e.g., 95%, it can be seen from Figure 13 that the position error rate of the fusion is equal to or below 32 cm/s, i.e., 32 cm per stride. This value improves the pocket INS’s and the foot INS’s in almost 10 cm/s. Although an improvement of 10 cm/s might not appear as a significant one, the effect accumulated over time is noticeable.

**Figure 14:**
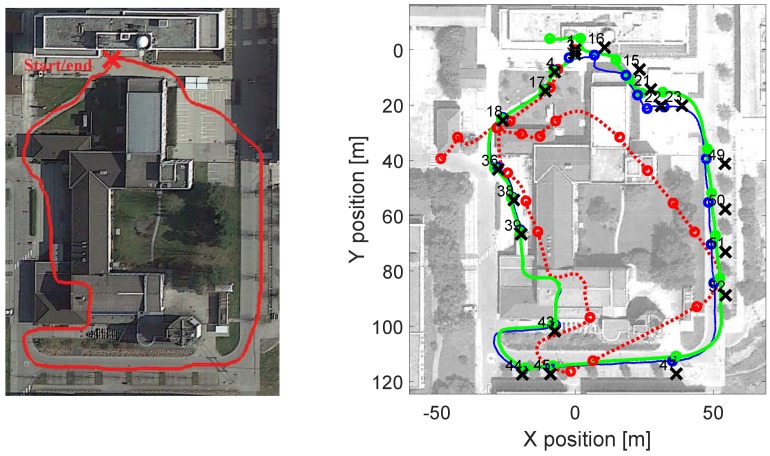
(**Left**) Approximate ground truth; (**Right**) Odometry estimated by each of the INS under evaluation. The dotted-red line is the pocket INS estimation. The thin-blue line is the foot INS estimation. The thick-green line is the loosely coupled fusion estimation. The cross marks indicate the ground truth points (GTP) visited during the walk. The circle marks indicate the estimated position of the GTPs.

Figure 14 is an example of a 5 min outdoor walk. It can be seen, on the right picture of Figure 14, that the foot INS is similar to the approximate ground truth depicted in the left picture of the same figure. The pocket INS, however, presents a higher drift in this particular walk. By visual inspection, one would choose the result of the foot INS. Figure 14 shows that the odometry estimated by the fusion is similar to the foot INS’s odometry. That is, the fusion is choosing the odometry with the least erroneous heading estimation, as one would do by comparing two or more odometries to the true walk.

**Figure 15:**
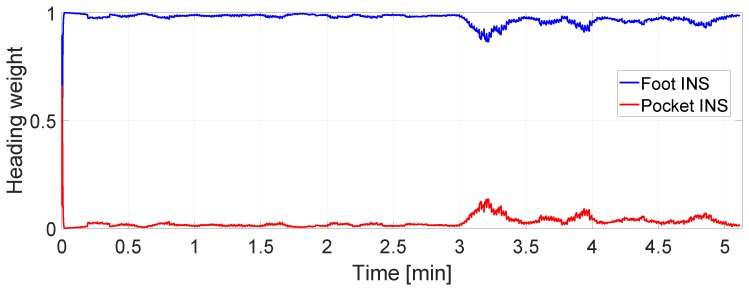
Heading weights estimated for the pocket INS and the foot INS in the walk depicted in Figure 14.

The improvement in the error of the loosely coupled fusion is mainly thanks to the heading weights, see Figure 15. The latter are estimated through the quality factor introduced in this work. Figure 15 shows that, according to the heading weights, the heading estimated by the foot INS is always better than the heading estimated by the pocket INS. The result is, thus, that the loosely coupled fusion will have the same heading as the foot INS.

## 4. Conclusions

This work explores the implementation of pedestrian position tracking by means of combining wearable-based INSs. In fact, this works proposes one method to combine two wearable-based INSs in a loosely coupled fashion. The original INSs are based on a foot-mounted IMU and a pocket-mounted IMU respectively. The results show that there is a benefit in combining wearable-based INSs to track a pedestrian’s position.

Two main conclusions can be extracted regarding pedestrian positioning. The first one is that the loosely coupled fusion here proposed profits, automatically, from the least erroneous information available at its input. This behaviour makes the loosely coupled fusion, in mean and standard deviation, better than either INS at its input. Furthermore, the position error rate of the loosely coupled fusion is 10 cm/s better than either the foot INS’s or pocket INS’s error rate in 95% of the cases. The beneficial effect of this result is especially noticeable in the long term.

The second main conclusion is that, it is possible to tell which INS is estimating a least erroneous heading without prior knowledge of the true walk performed. The determination of the heading quality can be done by means of only the inertial measurements and the orientation estimation of each INS.

## Figures and Tables

**Table 1:** Approaches to multiple inertial measurement unit (multi-IMU) navigation.

	Loose Coupling	Tight Coupling
**How?**	Deterministic Probabilistic	Constrained-based position tracking
**When?**	INSs work at different frequencies Only when estimates are good	
**Advantages**	Modularity Robustness	Efficient Less computational complexity
**Disadvantages**	Duplicated functionality	Requires all measurements

**Table 2:** Summary table of the experiments used in the evaluation.

Total No. of Walks	Total Time	Total Distance	Total No. GTPs
30	5 h	22.2 km	1125

**Table 3:** Mean (μ) and standard deviation (σ) of the position error rate of each INS under evaluation.

μ ± σ			
	**Pocket INS**	**Foot INS**	**Fusion**
$e_{P}$ **[m/s]**	0.16±0.12	0.13±0.19	0.12±0.10

## Abbreviations

The following abbreviations are used in this manuscript:

INS	Inertial Navigation System
IMU	Inertial Measurement Unit
RFID	Radio-Frequency IDentification
EKF	Extended Kalman Filter
RMSE	Root Mean Square Error
GTP	Ground Truth Point
